# Protective Effect of the Total Saponins from *Rosa laevigata* Michx Fruit against Carbon Tetrachloride-Induced Liver Fibrosis in Rats

**DOI:** 10.3390/nu7064829

**Published:** 2015-06-15

**Authors:** Deshi Dong, Lianhong Yin, Yan Qi, Lina Xu, Jinyong Peng

**Affiliations:** 1 College of Pharmacy, Dalian Medical University, No. 9 Western Lvshun South Road, Dalian 116044, China; E-Mails: deshidong@163.com (D.D.); yinlianhong1015@163.com (L.Y.); friendqy@163.com (Y.Q.); xulina627@163.com (L.X.); 2 The First Affiliated Hospital of Dalian Medical University, Dalian 116011, China

**Keywords:** protective effect, *Rosa laevigata* Michx, total saponins, liver fibrosis

## Abstract

In this study, the protective effect of the total saponins from *Rosa laevigata* Michx (RLTS) against liver fibrosis induced by carbon tetrachloride (CCl_4_) in rats was evaluated. The results showed that RLTS significantly rehabilitated the levels of alanine aminotransferase, aspartate aminotransferase, malondialdehyde, glutathione, glutathione peroxidase, catalase, superoxide dismutase, hydroxyproline, α-smooth muscle actin, collagen I, collagen III and fibronectin, which were confirmed using H&E, Sirius Red and Masson histopathological assays. Further research indicated that RLTS markedly reduced cytochrome P450 2E1 activity, attenuated oxidative stress, and suppressed inflammation. In addition, RLTS facilitated matrix degradation through down-regulation of matrix metalloproteinase2, matrix metalloproteinase 9 and metalloproteinases1, and exerted the anti-fibrotic effects through affecting transforming growth factor β/Smad, focal adhesion kinase/phosphatidylinositol-3-kinase/amino kinase terminal/70-kDa ribosomal S6 Kinase (FAK-PI3K-Akt-p70^S6K^) and mitogen-activated protein kinase (MAPK) signaling pathways. Taken together, our data indicate that RLTS can be applied as one effective candidate for the treatment of liver fibrosis in the future.

## 1. Introduction

Liver fibrosis, as the result of the excessive accumulation of extracellular matrix (ECM), is a typical chronic liver disease with significant mortality [1,2], in which the Kupffer cells release inflammatory mediators and free radicals [2] to cause oxidative stress and liver fibrogenesis [3]. In addition, hepatic stellate cells (HSCs) activation is a pivotal step in liver fibrosis. In this process, HSCs are activated from quiescent to myofibroblast-like cells caused by some factors including platelet derived growth factor (PDGF), tumor necrosis factor α (TNF-α), transforming growth factor β (TGF-β) or reactive oxygen species(ROS) [1,4]. This transdifferentiation is associated with several phenotypic alterations, including enhanced cell proliferation and contractility, increased cell migration and adhesion, α-smooth muscle actin (α-SMA) expression, acquisition of fibrogenic capacity and ECM overproduction [4,5].

Although liver fibrosis has been extensively studied, effective anti-fibrotic drugs are lacking at present. In recent years, herbal medicines with high efficacy and low toxicity have been paid more and more attention, in order to develop new and efficient drugs. Some active natural products including tetrahydrocurcumin, *Litchi chinensis* Sonn flower extract, sho-saiko-to, saponins from *Rhizoma* *panacis* Majoris and Panax notoginseng saponins have potent effects for the treatment of liver fibrosis [6,7,8,9,10,11].

*Rosa laevigata* Michx fruit has been consumed as medicine and food in China for many years to treat chronic cough, dermatologic disease, hyperpiesia, arterial sclerosis, urinary incontinence and menstrual irregularities [12,13]. The main active components of this plant are considered to be polysaccharose, flavonoids and saponins [14]. In our previous works, the significantly effects of the total saponins from *R*. *laevigata* Michx fruit (RLTS) against acute liver damages and non-alcoholic fatty liver disease have been reported [15,16,17]. However, the anti-fibrotic effect of the crude extract remains unknown to the best of our knowledge.

The aim of this study was to investigate the effects and possible mechanisms of RLTS against liver fibrosis induced by carbon tetrachloride (CCl_4_) in rats. Our findings suggest that supplementation of RLTS in the diet could be effective to treat hepatic fibrosis.

## 2. Materials and Methods

### 2.1. Materials and Reagents

*R. laevigata* Michx fruit was purchased from Yun-nan Qiancaoyuan Pharmaceutical Company Co., Ltd. (Kunming, China) and authenticated by Dr. Yunpeng Diao (College of Pharmacy, Dalian Medical University, Dalian, China). A voucher specimen (DLMU, JYZ-2012080426) was deposited in the Herbarium of College of Pharmacy, Dalian Medical University (Dalian, China). Silymarin (CAS: 65666-07-1, purity >98%) was obtained from Sigma Chemical Company (Milan, Italy). Alanine aminotransferase (ALT), aspartate aminotransferase (AST), hydroxyproline, malondialdehyde (MDA), glutathione peroxidase (GSH-Px), glutathione (GSH), catalase (CAT), superoxide dismutase (SOD) and bilirubin detection kits were provided by Nanjing Jiancheng Institute of Biotechnology (Nanjing, China). Enhanced BCA Protein Assay Kit was supplied by Beyotime Institute of Biotechnology (Jiangsu, China). Tissue Protein Extraction Kit was purchased from KeyGEN Biotech. CO., Ltd. (Nanjing, China). RNAiso Plus, PrimeScript^®^ RT reagent Kit with gDNA Eraser (Perfect Real Time) and SYBR^®^ Premix Ex Taq™ II (Tli RNaseH Plus) were obtained from TaKaRa Biotechnology Co., Ltd. (Dalian, China).

### 2.2. Preparation of the Crude Extract

The crude extract of the total saponins was prepared and determined with the methods reported in our previous study [15]. Briefly, the crude extract was extracted from *R. laevigata* Michx fruit by 75% ethanol solution with solid-liquid ratio (1:6, w/v) for 2 times (2.0 h for each). Then, the extract was separated by macroporous resin column chromatography. The solution of 30% aqueous ethanol was used to remove the unpurified chemicals, and 80% aqueous ethanol was used to elute the target chemicals, which was collected and dried to powder.

### 2.3. Chemical Determination

The content of the total saponins in the crude extract was determined by a colorimetric assay. The extract (200 μg) mixed with 5% vanillin-acetic acid (0.5 mL) and perchloric acid (1.5 mL) was incubated at 70 °C for 15 min. After cooling down in an ice bath, the solution was mixed with glacial acetic acid (total volume 10 mL), and the absorbance of the mixture was determined at 538 nm by a UV-Vis spectrophotometer (U-3010, Hitachi, Tokyo, Japan). The chemical oleanolic acid was used as the reference standard for external standard calibration [18]. The content of flavonoids in the extract was determined by a colorimetric method described in China Pharmacopeia [19] (pp.372–373) with some modification. Briefly, the solution of the extract was sequentially reacted with 5% (w/v) sodium nitrite, 10% (w/v) aluminum nitrate and 4% (w/v) NaOH. The absorbance was measured at 500 nm by UV-Vis spectrophotometer (U-3010, Hitachi), and rutin was used as the reference standard for external standard calibration [20].The quantitative method of tannin was colorimetric method described by China Pharmacopeia [19] (p. appendix 62) with minor modification. Briefly, the extract was reacted with phosphomolybdic tungstic acid and sodium carbonate, and the absorbance of the reaction product was detected at 760 nm. After the extract treated with casein, the non-absorbed polyphenol content was measured as mentioned above. Tannin content in the extract was calculated using a standard curve of gallic acid [21], and tannin content = total phenol content − non-adsorbed polyphenol content. To assay the content of organic acid in the extract, the sample (100 mg) was dissolved in 50 mL of aqueous solution and stirred bout 20 min. Two drops of phenolphthalein indicator solution were added into the mixture. With continuous stirring, 0.1 mol/L of NaOH standard solution was added until the color of the solution was changed to pink. The volume was obtained, and every 1 mL of NaOH standard solution (0.1 mol/L) was equivalent to 6.404 mg citric acid [19] (p. 29), and then the content of organic acid in the extract was calculated.

### 2.4. Animals and Experimental Design

Male Sprague-Dawley (SD) rats, weighing 160–180 g, were provided by the Experimental Animal Center of Dalian Medical University, Dalian, China (Quality certificate number: SCXK (Liao) 2013-0003). Animals used in the experiment were approved by the Ethical Committee and the Laboratory Animal Center of Dalian Medical University according to the China National Institutes of Health Guidelines for the Care and Use of Laboratory Animals (approval number: SYXK (Liao) 2013-0006; 20 May 2013). The rats were kept under standard condition with a temperature of 25 ± 2 °C, relative humidity of 55% ± 5%, room air changes 12–18 times/h, and a 12 h light/dark cycle. After acclimatization for one week, the animals were randomly divided into seven groups consisting of 10 rats per group. The rats in Group I (RLTS control) were orally administered 210 mg/kg RLTS suspended in 0.5% carboxymethylcellulose sodium (CMC-Na) and in Group II (normal control) were given vehicle (0.5% CMC-Na) only. RLTS control and normal control were given olive oil (2 mL/kg) intraperitoneally twice a week. The rats in Group III (model) were administered CCl_4_ dissolved in olive oil (2 mL/kg, 50% v/v), intraperitoneally. The animals in Groups IV–VI were administrated CCl_4_ and RLTS at the doses of 70, 140 and 210 mg/kg. The rats in Group VII were given CCl_4_ and 210 mg/kg of silymarin. The CCl_4_ was administered twice a week for 10 weeks. RLTS was administered daily for 10 weeks. At the end of 10 weeks, animals were fasted overnight and then anesthetized with ether. Blood and liver samples were collected for further analysis.

### 2.5. Pathological Examination

A portion of liver was fixed in 10% neutral buffered formalin solutions for 24 h, processed by standard histological procedures, followed by embedding in paraffin and cut into 5 μm sections. The samples were stained with hematoxylin and eosin (H&E) and evaluated using a microscope (Leica DM4000B, Solms, Germany). The fibrosis changes were evaluated by staining with Masson’s trichrome and Sirius red, and the quantitative assays were performed using the image software according to the instructions.

### 2.6. Transmission Electron Microscopy (TEM)

Fresh liver tissue (~1 mm^3^) obtained from the rats in control, model and RLTS (210 mg/kg) group were fixed in 2.5% glutaraldehyde at 4 °C overnight. The samples were treated as previously described [22]. The ultramicrotomies were stained and the images were photographed with an electron microscope (JEM-2000EX, JEDL, Sagamihara, Japan).

### 2.7. Biochemical Analysis

The activities of aspartate aminotransferase (AST), alanine aminotransferase (ALT) and bilirubin content in serum, and the levels of glutathione peroxidase (GSH-Px), glutathione (GSH), catalase (CAT), superoxide dismutase (SOD) and malondialdehyde (MDA) in liver were determined by detection kits in the light of the manufacturer’s instructions. Hepatic hydroxyproline content was assayed colorimetrically according to the instruction manual.

### 2.8. Immunofluorescence and Immunohistochemical Assays for Alpha-Smooth Muscle Actin (α-SMA) and Transforming Growth Factor-β1 (TGF-β1)

The paraffin sections were deparaffinized and rehydrated in a graded series of ethanol, then treated with 0.01 mol/L citrate (pH = 6.0) in a microwave oven for 15 min. After that, the sections were incubated in a solution of 3% hydrogen peroxide for 10 min at room temperature, followed by blocking nonspecific protein with normal goat serum for 20 min. The treated slides were incubated at 4 °C overnight with rabbit anti-α-SMA (1:100, dilution) or rabbit anti-TGF-β1 (1:100, dilution), then followed by incubation with Cy3-conjugated goat anti-rabbit IgG for 30 min, or biotinylated goat anti-rabbit IgG and horseradish peroxidase-conjugated streptavidin for 15 min. The slides were washed with PBS and counterstained with 4’,6-diamidino-2-phenylindole (DAPI) or 3,3’-diaminobenzidine (DAB) and hematoxylin. The images were obtained by a fluorescent microscopy (Olympus BX63, Olympus, Tokyo, Japan) or a light microscope (Leica DM4000B).

### 2.9. Quantitative Real-Time PCR Assay

Total RNA was isolated from liver tissues using RNAiso Plus reagent according to the supplier’s instruction. Amplification of cDNA and quantitative real-time PCR analyses were carried out as described previously [23]. The forward and reverse primers used for real-time PCR assay are presented in Table 1.

**Table 1:** The primer sequences used for real-time PCR assay in rats.

Gene	Full Name of the Gene	Sequence (5′→3′) ^a^	GenBank ^b^
GAPDH	Glyceraldehyde 3-phosphate	GGCACAGTCAAGGCTGAGAATG	NM_017008.3
	dehydrogenase	ATGGTGGTGAAGACGCCAGTA	
Col 1A1	Collagen I	GACATGTTCAGCTTTGTGGACCC	NM_053304
		AGGGACCCTTAGGCCATTGTGTA	
Col 3A1	Collagen III	TTTGGCACAGCAGTCCAATGTA	NM_032085
		GACAGATCCCGAGTCGCAGA	
TNF-α	Tumor necrosis factor α	TCAGTTCCATGGCCCAGAC	NM_012675.3
		GTTGTCTTTGAGATCCATGCCATT	
IL-1β	Interleukin 1β	CCCTGAACTCAACTGTGAAATAGCA	NM_031512.2
		CCCAAGTCAAGGGCTTGGAA	
IL-6	Interleukin 6	ATTGTATGAACAGCGATGATGCAC	NM_012589.1
		CCAGGTAGAAACGGAACTCCAGA	

^a^ Shown as forward primer followed by reverse primer; ^b^ GenBank accession number.

### 2.10. Western Blotting Assay

Total protein was isolated from liver tissue using the tissue protein extraction kit and the protein concentration was measured by BCA Protein Assay Kit. Sample protein was separated using sodium dodecyl sulfate-polyacrylamide gel electrophoresis (SDS-PAGE) with 8%, 10% or 12% separating gel and the electrophoresis system. The proteins in the gel were then transferred onto a polyvinylidene fluoride (PVDF) membrane. After transfer, the membrane was blocked by 5% dried skim milk for 3 h. The proteins were probed with the primary antibodies (Table 2) at 4 °C overnight and followed by probing with the goat anti-rabbit (1:2500 dilution) IgG-horseradish peroxidase-conjugated secondary antibody at room temperature for 2 h. Protein expression was detected using an enhanced chemiluminescence (ECL) method and the images were captured by Bio-spectrum Gel Imaging System (UVP, Upland, CA, USA). The expression of protein was quantified using Gel-Pro Analyzer software and the changes in expression were normalized to the GAPDH control.

**Table 2:** The information of the antibodies used in the present work.

Antibody	Full Name	Source	Dilution	Company
Fibronectin	Fibronectin	rabbit	1:500	Proteintech Group, Chicago, IL, USA
Col 1A1	Collagen I	rabbit	1:500	Bioworld Technology, St. Louis Park, MN, USA
Col 3A1	Collagen III	rabbit	1:500	Bioworld Technology, St. Louis Park, MN, USA
MMP-2	Matrix metalloproteinase 2	rabbit	1:500	Proteintech Group, Chicago, IL, USA
MMP-9	Matrix metalloproteinase 9	rabbit	1:500	Proteintech Group, Chicago, IL, USA
TIMP1	Tissue inhibitors of metalloproteinases 1	rabbit	1:1000	Proteintech Group, Chicago, IL, USA
p-ERK	Phosphorylated-Extracellular regulated kinase	rabbit	1:500	Bioworld Technology, St. Louis Park, MN, USA
ERK	Extracellular regulated kinase	rabbit	1:500	Bioworld Technology, St. Louis Park, MN, USA
p-p38	Phosphorylated-p38 mitogen-activated protein kinase	rabbit	1:500	Bioworld Technology, St. Louis Park, MN, USA
p38	p38 mitogen-activated protein kinase	rabbit	1:500	Bioworld Technology, St. Louis Park, MN, USA
p-JNK	Phosphorylated-c-Jun *N*-terminal kinase	rabbit	1:500	Bioworld Technology, St. Louis Park, MN, USA
JNK	c-Jun *N*-terminal kinase	rabbit	1:500	Bioworld Technology, St. Louis Park, MN, USA
HO-1	Heme oxygenase-1	rabbit	1:1000	Bioworld Technology, St. Louis Park, MN, USA
SOD2	Superoxide dismutase 2	rabbit	1:1000	Proteintech Group, Chicago, IL, USA
Nrf2	Nuclear factor erythroid 2-related factor 2	rabbit	1:1000	Bioworld Technology, St. Louis Park, MN, USA
CYP2E1	Cytochrome P450 2E1	rabbit	1:1000	Proteintech Group, Chicago, IL, USA
p-Smad2/3	Phosphorylated-Smad 2/3	rabbit	1:1000	Bioworld Technology, St. Louis Park, MN, USA
Smad2/3	Smad 2/3	rabbit	1:1000	Proteintech Group, Chicago, IL, USA
Smad7	Smad 7	rabbit	1:1000	Abcam, Cambridge, MA, USA
PDGF-β	Platelet derived growth factor-β	rabbit	1:1000	Proteintech Group, Chicago, IL, USA
p-Akt	Phosphorylated-amino kinase terminal	rabbit	1:500	Proteintech Group, Chicago, IL, USA
Akt	Amino kinase terminal	rabbit	1:500	Proteintech Group, Chicago, IL, USA
p-p70S6K	Phosphorylated-70-kDa ribosomal S6 Kinase	rabbit	1:1000	Santa Cruz Biotechnology, Santa Cruz, CA, USA
p70S6K	70-kDa ribosomal S6 Kinase	rabbit	1:1000	Santa Cruz Biotechnology, Santa Cruz, CA, USA
TLR4	Toll-like receptor 4	rabbit	1:500	Proteintech Group, Chicago, IL, USA
MyD88	Myeloid differentiation factor 88	rabbit	1:1000	Santa Cruz Biotechnology, Santa Cruz, CA, USA
NF-κB	Nuclear factor kappa B	rabbit	1:1000	Proteintech Group, Chicago, IL, USA
iNOS	Inducible nitric oxide synthase	rabbit	1:1000	Proteintech Group, Chicago, IL, USA
COX-2	Cyclooxygenase-2	rabbit	1:1000	Proteintech Group, Chicago, IL, USA
IL-10	Interleukin-10	rabbit	1:400	Boster Biological Technology, Wuhan, China
TNF-α	Tumor necrosis factor α	rabbit	1:400	Proteintech Group, Chicago, IL, USA
IL-1β	Interleukin-1β	rabbit	1:500	Bioworld Technology, St. Louis Park, MN, USA
IL-6	Interleukin-6	rabbit	1:500	Bioworld Technology, St. Louis Park, MN, USA
GAPDH	Glyceraldehyde 3-phosphate dehydrogenase	rabbit	1:1000	Proteintech Group, Chicago, IL, USA

### 2.11. Statistical Analysis

Data from each group were expressed as mean and standard deviation (SD). Statistical comparison between groups was done using one-way ANOVA followed by Tukey *post hoc* test, using *p* < 0.05 and *p* < 0.01 as the level of significance.

## 3. Results

### 3.1. Contents of the Chemicals in the Extract

The chemicals of saponins, flavonoids, tannin and organic acid are considered to be the main components in the extract from *R. laevigata* Michx fruit [24], which were all determined in the present work. The detection wavelengths of saponins, flavonoids and tannin were optimized (shown in Supplementary Figure S1). The calibration curves were plotted with a series of concentrations of oleanolic acid, gallic acid and rutin with the regression equations in the form of *Y* = a*X* + b, where *X* and *Y* are the concentrations of the standards and the absorptions. The average contents of total saponins, total flavonoids, tannin and organic acid in the crude extract were 71.3%, 16.8%, 8.6% and 2.7% (*n* = 3). Thus, the total content of the known chemicals in the extract was reached to 99.4%, and the total saponins from *R. laevigata* Michx fruit (RLTS) was the major chemicals in the extract.

### 3.2. RLTS Improved Liver Injury Caused by CCl_4_

**Figure 1:**
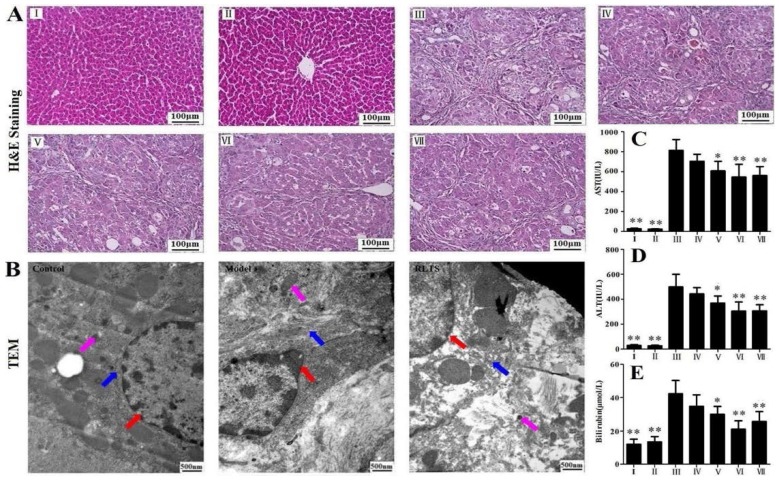
Effects of *Rosa laevigata* Michx fruit (RLTS) on histopathological examination based on H&E staining (**A**); Effect of RLTS against CCl_4_-induced liver fibrosis based on transmission electron microscopy (TEM) assay (magnification, 25,000×). The red, blue and pink arrows indicate nuclei, endoplasmic reticulum and mitochondria, respectively (**B**); Effects of RLTS on serum ALT, AST activities and the bilirubin content (**C**–**E**). (**I**) RLTS control; (**II**) Normal control; (**III**) model; (**IV**) RLTS (70 mg/kg) + CCl_4_; (**V**) RLTS (140 mg/kg) + CCl_4_; (**VI**) RLTS (210 mg/kg) + CCl_4_; (**VII**) silymarin (210 mg/kg) + CCl_4_. Values are expressed as mean ± SD (*n* = 10). * *p* < 0.05; ** *p* < 0.01 *vs.* model group.

As shown in Figure 1A, the liver from the model group showed extensive changes including marked necrosis, hepatocyte ballooning and infiltration of inflammatory cells, but the changes were obviously attenuated by RLTS. As shown in Figure 1B, irregular nuclei, dilated endoplasmic reticulum, swollen mitochondria and chromatin condensation were found in model group. However, RLTS treatment significantly attenuated the changes caused by CCl_4_. As shown in Figure 1C–E, significant increased levels of ALT, AST and bilirubin were observed in model group, which were all markedly reduced by RLTS or silymarin.

### 3.3. Effects of RLTS on Liver Fibrosis Caused by CCl_4_ in Rats

**Figure 2:**
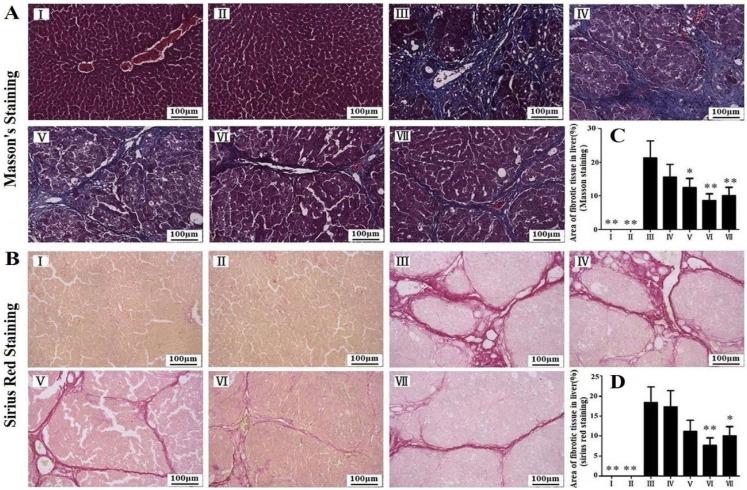
Effects of *Rosa laevigata* Michx fruit (RLTS) against CCl_4_-induced liver fibrosis in rats based on Masson staining and Sirius Red staining (**A**,**B**); Statistic analysis of Masson staining and Sirius red staining (**C**,**D**). (**I**) RLTS control; (**II**) Normal control; (**III**) model; (**IV**) RLTS (70 mg/kg) + CCl_4_; (**V**) RLTS (140 mg/kg) + CCl_4_; (**VI**) RLTS (210 mg/kg) + CCl_4_; (**VII**) silymarin (210 mg/kg) + CCl_4_. Values are expressed as mean ± SD (*n* = 4). * *p* < 0.05; ** *p* < 0.01 *vs.* model group.

As shown in Figure 2A,B, prolonged CCl_4_ treatment induced obvious accumulation of collagen in the liver based on Masson and Sirius Red staining in model group. Treatment with RLTS (210 mg/kg) resulted in the significantly decreased accumulation of collagen in the liver compared with model group (Figure 2C,D).

**Figure 3:**
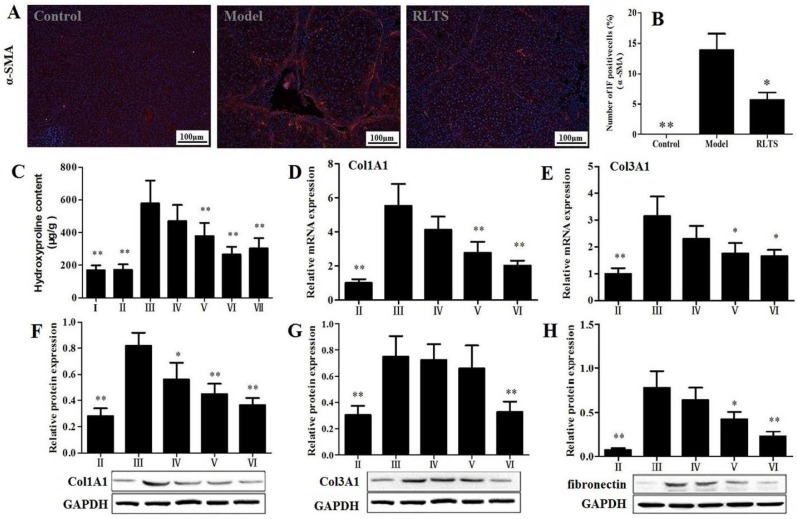
Effect of *Rosa laevigata* Michx fruit (RLTS) (210 mg/kg) on the expression of α-SMA based on immunofluorescence assay (magnification, 100×) (**A**); Statistical analysis of the fluorescence intensity (**B**); Effect of RLTS on hepatic hydroxyproline content (**C**); Effect of RLTS on the gene levels of collagen I and collagen III (**D**–**E**); Effect of RLTS on the protein expressions of collagen I, collagen III and fibronectin (**F**–**H**). (**I**) RLTS control; (**II**) Normal control; (**III**) model; (**IV**) RLTS (70 mg/kg) + CCl_4_; (**V**) RLTS (140 mg/kg) + CCl_4_; (**VI**) RLTS (210 mg/kg) + CCl_4_; (**VII**) silymarin (210 mg/kg) + CCl_4_. Values are expressed as mean ± SD (*n* = 4). * *p* < 0.05; ** *p* < 0.01 *vs.* model group.

The effect of RLTS on α-SMA expression was investigated (Figure 3A). As expected, strongly positive cells were observed in model group, and RLTS significantly decreased the positive cells. As shown in Figure 3B, the declined level in RLTS-treated group (210 mg/kg) was reached to 31.42% ± 7.24% (*n* = 3) compared with model group. As shown in Figure 3C, CCl_4_ administration significantly increased the concentration of hydroxyproline compared with normal group (*p* < 0.01). RLTS treatment significantly reduced hydroxyproline level in a dose-dependent manner by 18.85%, 34.75% and 47.91% at the doses of 70, 140, 210 mg/kg compared with model group. The gene or protein expression levels of collagen I, collagen III and fibronectin were increased in model group (Figure 3D–H), which were significantly down-regulated by RLTS (210 mg/kg) to 2.73-, 1.90-, 2.25-, 2.29- and 3.39-fold, respectively, compared with model group.

### 3.4. RLTS Decreased the Levels of Pro-Fibrotic Factors Related to ECM

**Figure 4:**
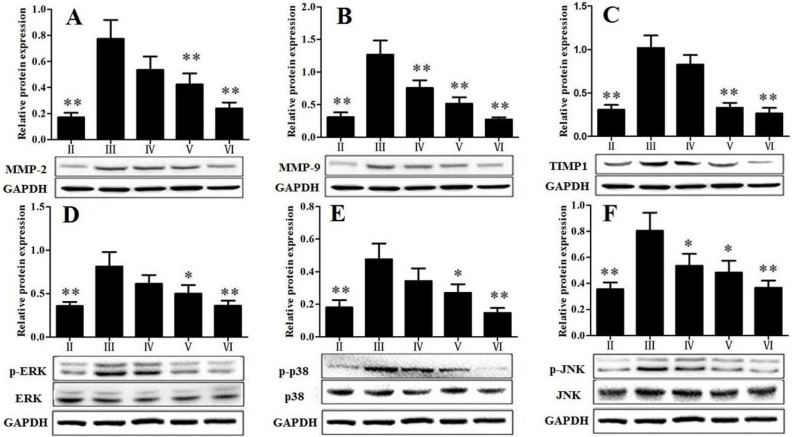
Effect of *Rosa laevigata* Michx fruit (RLTS) on the protein expressions of MMP-2 (**A**); MMP-9 (**B**); TIMP1 (**C**); p-ERK (**D**); p-p38 (**E**) and p-JNK (**F**). (**II**) Normal control; (**III**) model; (**IV**) RLTS (70 mg/kg) + CCl_4_; (**V**) RLTS (140 mg/kg) + CCl_4_; (**VI**) RLTS (210 mg/kg) + CCl_4_. Values are expressed as mean ± SD (*n* = 4). * *p* < 0.05; ** *p* < 0.01 *vs.* model group.

As shown in Figure 4A–C, the protein levels of matrix metalloproteinase 2 (MMP-2), matrix metalloproteinase 9 (MMP-9) and tissue inhibitors of metalloproteinases1 (TIMP1) were increased in the rats chronically administered with CCl_4_ (*p* < 0.01). In the group treated with RLTS (210 mg/kg), the levels of these pro-fibrotic factors were significantly down-regulated by 3.22-, 4.64- and 3.87-fold, respectively, compared with model group.

### 3.5. Effect of RLTS on MAPK Signal Pathway

As shown in Figure 4D–F, the results showed that CCl_4_ administration increased the levels of phosphorylated extracellular regulated kinase 1/2 (ERK1/2), c-Jun *N*-terminal kinase (JNK), and p38 mitogen-activated protein kinase (p38), and RLTS (210 mg/kg) significantly decreased the expressions by 2.26-, 2.19- and 3.25-fold, respectively, compared with the model group.

### 3.6. RLTS Reduced Lipid Peroxidation and Oxidative Stress

**Figure 5:**
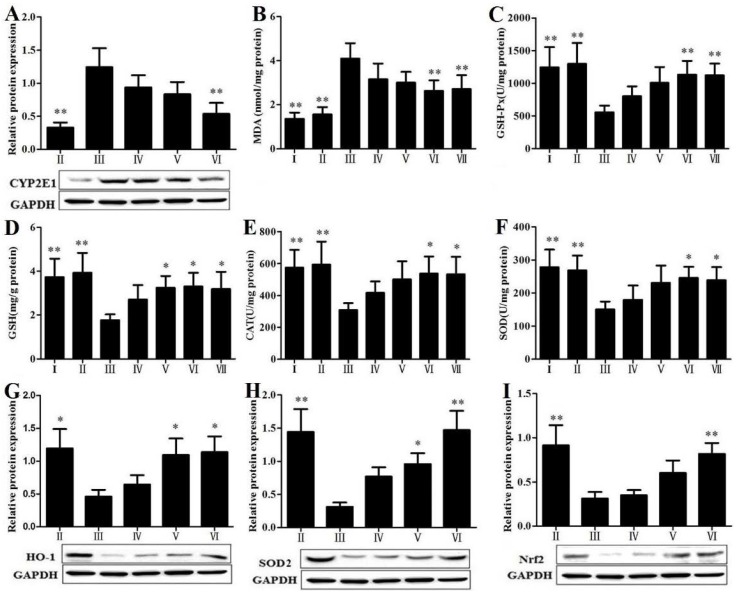
Effect of *Rosa laevigata* Michx fruit (RLTS) on the protein expression of CYP2E1 (**A**); Effects of RLTS on the levels of MDA, GSH-Px, GSH, CAT and SOD in livers (**B**–**F**); Effects of RLTS on the protein expressions of HO-1, SOD2 and Nrf2 (**G**–**I**). (**I**) RLTS control; (**II**) Normal control; (**III**) model; (**IV**) RLTS (70 mg/kg) + CCl_4_; (**V**) RLTS (140 mg/kg) + CCl_4_; (**VI**) RLTS (210 mg/kg) + CCl_4_; (**VII**) silymarin (210 mg/kg) + CCl_4_. Values are expressed as mean ± SD (*n* = 4). * *p* < 0.05; ** *p* < 0.01 *vs.* model group.

As shown in Figure 5A, the protein level of cytochrome P450 2E1 (CYP2E1) was significantly suppressed by RLTS with a dose-dependent manner. Our findings showed that RLTS was able to counter the CCl_4_-induced oxidative stress and suppressed oxidative liver injury. As shown in Figure 5B–F, in model group, CCl_4_-induced liver fibrosis provoked a significant promotion of liver MDA content and obvious reductions of liver GSH-Px, GSH, CAT and SOD activities compared with normal group. Our results showed that RLTS dramatically decreased liver MDA level and markedly increased the activities of GSH-Px, GSH, CAT and SOD. As shown in Figure 5G–I, compared with control group, the levels of heme oxygenase-1 (HO-1), superoxide dismutase 2 (SOD2) and nuclear factor erythroid 2-related factor 2 (Nrf2) were markedly decreased in model group. Their expressions in RLTS (210 mg/kg) group were markedly up-regulated by 2.47-, 4.69-, 2.60-fold, respectively, compared with the model group.

### 3.7. RLTS Decreased Activation, Proliferation and Migration of HSCs

**Figure 6:**
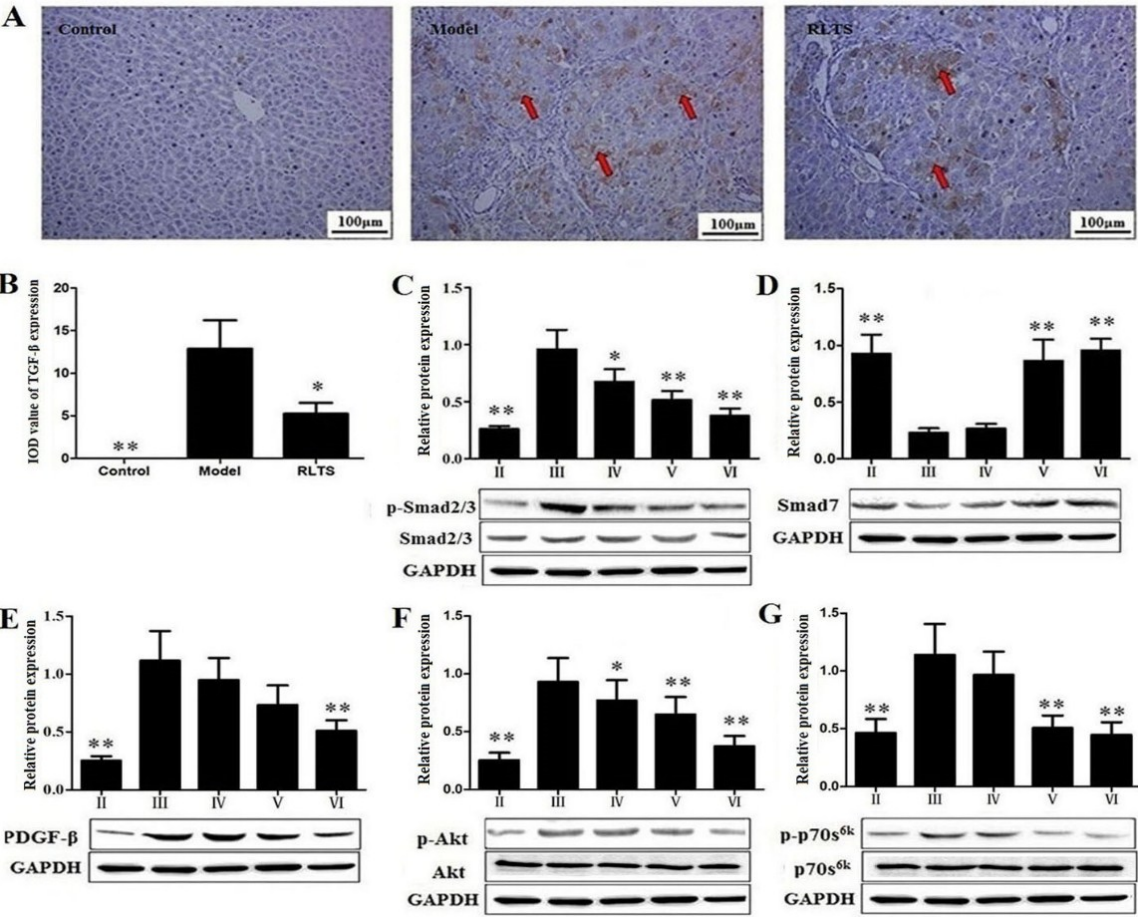
Effect of *Rosa laevigata* Michx fruit (RLTS) (210 mg/kg) on the expression of TGF-β1 based on immunohistochemical assay (magnification, 100×) (**A**); Statistical analysis of the integrated optical density (**B**); Effect of RLTS on the expressions of p-Smad2/3, Smad7, PDGF-β, p-Akt and p-p70^S6K^ (**C**–**G**). (**II**) Normal control; (**III**) model; (**IV**) RLTS (70 mg/kg) + CCl_4_; (**V**) RLTS (140 mg/kg) + CCl_4_; (**VI**) RLTS (210 mg/kg) + CCl_4_. Values are expressed as mean ± SD (*n* = 4). * *p* < 0.05, ** *p* < 0.01 *vs.* model group.

The effect of RLTS on TGF-β1 expression was investigated by immunohistochemical assay. As shown in Figure 6A,B, the positive area of TGF-β1 was observed in rats of CCl_4_-administration group, and RLTS administration significantly decreased the positive area by 34.35% ± 6.87% (*n* = 3), compared with model group. As shown in Figure 6C,D, the expression level of p-Smad2/3 in CCl_4_-treated group was strongly up-regulated by 3.69-fold, while Smad7 was down-regulated by 4.02-fold, which were all significantly reversed by RLTS. In addition, RLTS treatment effectively reduced PDGF-β expression, amino kinase terminal (Akt) and 70-kDa ribosomal S6 Kinase (p70^S6K^) activations by 2.19-, 2.50-, 2.57-fold, respectively, compared with the model group (Figure 6E–G).

### 3.8. Effects of RLTS on the Protein Expressions Related to Inflammatory Mediators

**Figure 7:**
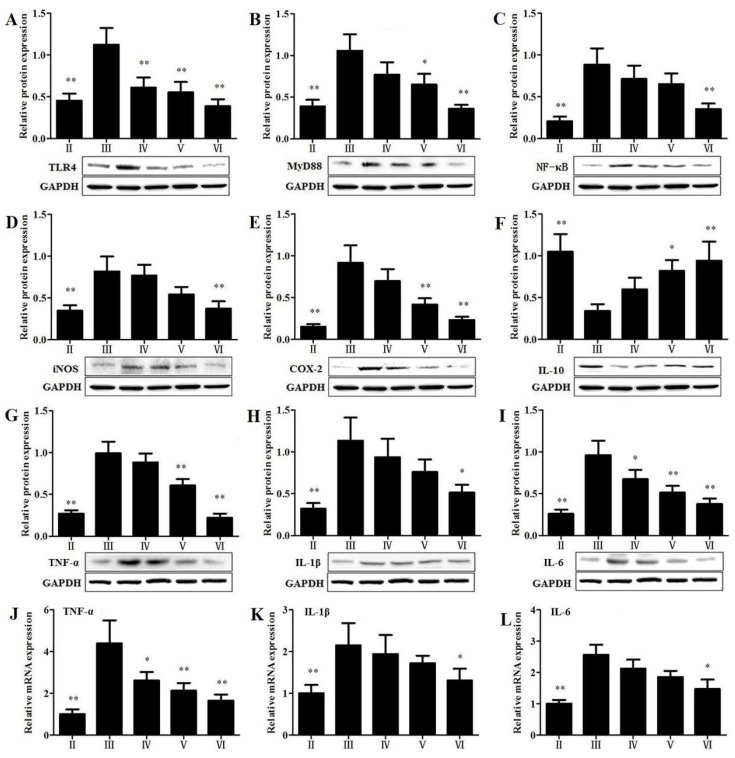
Effects of *Rosa laevigata* Michx fruit (RLTS) on the expressions of TLR4, MyD88, NF-κB, iNOS, COX-2, IL-10, TNF-α, IL-1β and IL-6 (**A–L**). (**II**) Normal control; (**III**) model; (**IV**) RLTS (70 mg/kg) + CCl_4_; (**V**) RLTS (140 mg/kg) + CCl_4_; (**VI**) RLTS (210 mg/kg) + CCl_4_. Values are expressed as mean ± SD (*n* = 4). * *p* < 0.05, ** *p* < 0.01 *vs.* model group.

As shown in Figure 7, the protein levels of toll-like receptor 4 (TLR4), myeloid differentiation factor 88 (MyD88), nuclear factor kappa B (NF-κB), iNOS, cyclooxygenase-2 (COX-2), TNF-α, interleukin-1β (IL-1β) and interleukin-6 (IL-6) in model group were markedly increased by 2.48-, 2.71-, 4.27-, 2.35-, 6.11-, 3.67-, 3.54- and 3.69-fold, respectively, compared with control group (*p* < 0.01). In the RLTS treatment group (210 mg/kg), the levels of these proteins were markedly reduced. Similarly, RLTS at the dose of 210 mg/kg down-regulated the gene levels of TNF-α, IL-1β and IL-6 by 2.66-, 1.65- and 1.75-fold, respectively, compared with model group (*p* < 0.05). In contrast, the level of interleukin-10 (IL-10) was decreased by 3.08-fold in model group compared with control group, which was significantly elevated by RLTS.

## 4. Discussion

In this study, chronic CCl_4_ administration caused classical fibrosis or early cirrhosis, and severe impairment of liver function was evidenced by the elevated serum levels of ALT, AST and total bilirubin [25], which were all markedly restored by RLTS, suggesting the potent effects of the natural extract RLTS against liver fibrosis in rats induced by CCl_4_.

Liver fibrosis occurs when there is an excessive accumulation of ECM, which can be caused by an imbalance between excess synthesis of fibrillar components including collagen I, collagen III and fibronectin [26,27]. During fibrosis, endopetidases including matrix metalloproteinases (MMPs), especially MMP-2 and MMP-9, are up-regulated and involved in the degradation and formation of ECM [26]. In addition, the tissue inhibitors of metalloproteinases (TIMPs) are also up-regulated by activated HSCs [28]. Consequently, though MMPs are present, but their actions are suppressed by high levels of TIMPs [28]. In the present work, the results, as we expected, showed that the up-regulations of collagen I, collagen III, fibronectin, MMP-2, MMP-9 and TIMP1 caused by CCl_4_ were evidently decreased by RLTS. Moreover, the level of hydroxyproline, an important biomarker of liver fibrosis, was effectively decreased by RLTS. In agreement with these results, RLTS significantly ameliorated CCl_4_-induced liver fibrosis and pathological tissue damage based on H&E, Masson and Sirius red staining. CCl_4_ is metabolized by CYP2E1 in liver cells to produce trichloromethyl radical (CCl_3_•), trichloromethyl peroxyl radical (CCl_3_OO•) and ROS, which can cause liver damage and increase the production of fibrotic tissue [29,30]. In the current study, RLTS pretreatment markedly decreased CYP2E1 expression, which implied that the formation of these radicals and ROS were decreased, thereby attenuated liver fibrosis. In addition, CCl_4_ administration significantly decreased the levels of GSH-Px, GSH, CAT and SOD in livers, implying that the defense systems were failed against oxidative stress. RLTS treatment was found to evidently elevate the levels of these antioxidants, which indicated that the extract counteracted the elevated formation of free radicals and ROS induced by CCl_4_, therefore decreased oxidative stress. Moreover, the increased MDA content in liver suggested the failed against antioxidant defense to prevent the formation of excessive free radicals and enhanced lipid peroxidation leading to tissue damage [31]. RLTS significantly decreased MDA level, to enhance the antioxidant defenses. Next, the protein levels of HO-1, SOD2 and Nrf2 in model group were significantly decreased (*p* < 0.05), compared with control group, which were elevated by RLTS. Taken together, our findings revealed that RLTS markedly decreased oxidative stress to inhibit HSCs activation as well as suppress liver fibrosis.

HSCs activation plays a central role in liver fibrosis [32]. The elevation of α-SMA is a marker of phenotypic transformation of HSCs into myofibroblast-like cells. In the present work, RLTS treatment significantly reduced α-SMA expression compared with model group, indicating that RLTS might deactivate HSCs. TGF-β, one of the most important cytokines involved in fibrosis, is found to be released by the activated hepatocytes during liver injury and in turn activates HSCs [2,33]. Smad2 and Smad3, two TGF-β receptor substrates, can form a complex with Smad4, and then translocate into the nucleus and regulate transcription of target genes [34]. Smad7 is an inhibitor of Smad signaling and effectively inhibits Smad2/3 activation and subsequent downstream signaling events [35]. In our work, RLTS markedly reduced the expressions of TGF-β and the activated Smad2/3, and increased the expression of Smad7, thus suppressed HSCs activation. Namely, the effect of RLTS against liver fibrosis maybe affect TGF-β/Smad signaling pathway.

PDGF, the most potent mitogenic and proliferative cytokine for HSCs, is up-regulated during HSCs activation and can be regulated by TGF-β [34]. PDGF has been proven to activate focal adhesion kinase (FAK) in fibrotic rat livers [36]. Activation of FAK subsequently causes the activation of several downstream kinases such asPI3K, Akt and p70^S6K^ [34]. Our findings showed that RLTS treatment effectively decreased the expression of PDGF-β and the activation of Akt and p70^S6K^, suggesting that the inhibitory effects of RLTS on HSCs activation might be related to affecting PDGF level and ultimately adjusting FAK-PI3K-Akt-p70^S6K^ signaling pathway.

The mitogen-activated protein kinase (MAPK) signaling pathway can be stimulated by activated HSCs [34]. ERK, JNK and p38 are the main members of the family. When MAPKs are activated, they can translocate into the nucleus and activate several transcription factors, leading to various cellular responses such as proliferation, differentiation and regulation of specific metabolic pathways [37,38]. Szuster-Ciesielska *et al.*, confirmed that phosphorylation of p38 and ERK are involved in HSCs activation, and JNK activation occurs concomitantly with enhanced HSCs migratory activity [39]. In the current study, the increased levels of phosphorylated ERK1/2, p38 and JNK were observed after CCl_4_ administration, which were significantly reversed by RLTS. These findings suggested that the effect of RLTS against liver fibrosis may suppress HSCs activation through MAPK signaling pathway.

**Figure 8:**
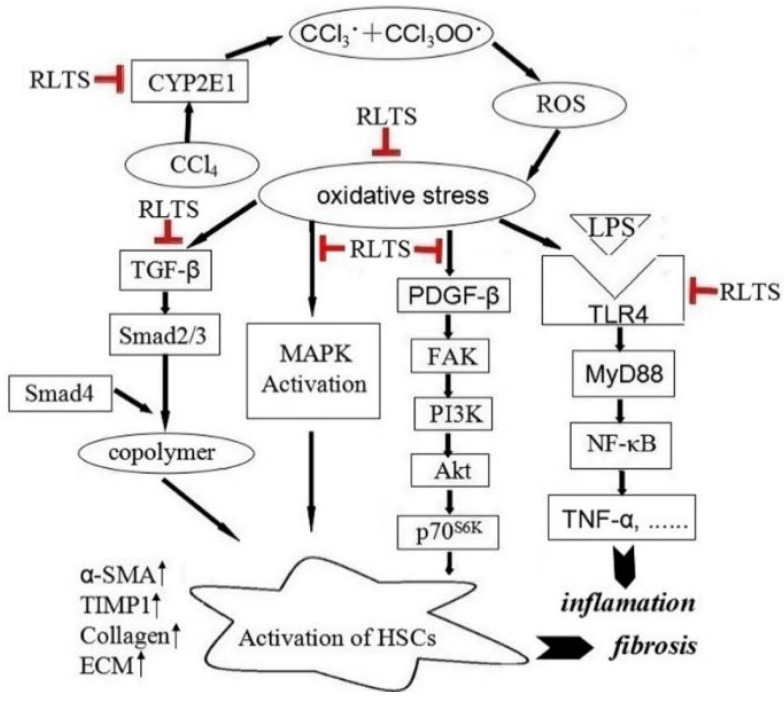
*Rosa laevigata* Michx fruit (RLTS) down-regulated collagen expression, reduced extracellular matrix (ECM) accumulation, decreased CYP2El activity, attenuated oxidative stress, suppressed inflammation, inhibited hepatic stellate cells (HSCs) activation via TGF-β/Smad, FAK-PI3K-Akt-p70^S6K^ and MAPK signaling pathways.

It is well known that the inflammatory response takes part in collagen synthesis and accumulation [33]. Fibrosis represents the final common pathway of chronic hepatic inflammation though the constituents of inflammation vary in different liver diseases [40]. After liver injury, the level of lipopolysaccharide (LPS) increases to enhance hepatic fibrosis [41]. Toll-like receptors (TLRs) are the family of pattern recognition receptors that sense conserved both pathogen and damage-associated molecular patterns [42]. TLR4 is a member of the TLRs family and a receptor for LPS, has been found to be closely related to liver fibrosis [43]. TLR4 plays a critical role in innate immunity by provoking inflammatory responses [44]. TLR4 activation can stimulate adapter proteins including MyD88 [45], to activate NF-κB [46].Activated NF-κB modulate proinflammatory mediators including TNF-α, IL-1β and IL-6 [47], while TNF-α can increase the expression of COX-2 [48]. Furthermore, IL-1 and TNF-α regulate the expression of iNOS [49]. In the present paper, the levels of TLR4, MyD88, NF-κB, iNOS, COX-2, TNF-α, IL-1β and IL-6 associated with inflammation in CCl_4_-treated group were significantly increased compared with control group. Moreover, the expression of anti-inflammatory mediator IL-10 was markedly decreased in model group. However, RLTS significantly down-regulated the levels of pro-inflammatory cytokines and up-regulated the level of anti-inflammatory cytokine to decrease inflammatory milieu and inhibit the activation of HSCs. These data suggested that the anti-fibrosis effect of RLTS may affect inflammation through TLR4/MyD88/NF-κB signaling pathway (as presented in Figure 8).

## 5. Conclusions

The present study showed that RLTS was effective on attenuating CCl_4_-induced liver fibrosis in rats through modulating inflammatory process and oxidative stress, regulating TGF-β/Smad, FAK-PI3K-Akt-p70^S6K^ and MAPK signaling pathways to restrain HSCs activation and decrease ECM production. This natural extract could be developed as an effective food and health care product for the prevention of liver fibrosis in the future.

## Supplementary Files

Supplementary File 1
