# Supplementary material for: Protective Effect of the Total Saponins from Rosa laevigata Michx Fruit against Carbon Tetrachloride-Induced Liver Fibrosis in Rats
Source: Nutrients. 2015 Jun 15;7(6):4829–50. doi: 10.3390/nu7064829 (PMC4488818; doi:10.3390/nu7064829)
Supplement: Supplementary File 1 [file nutrients-07-04829-s001.docx]

Supporting Materials


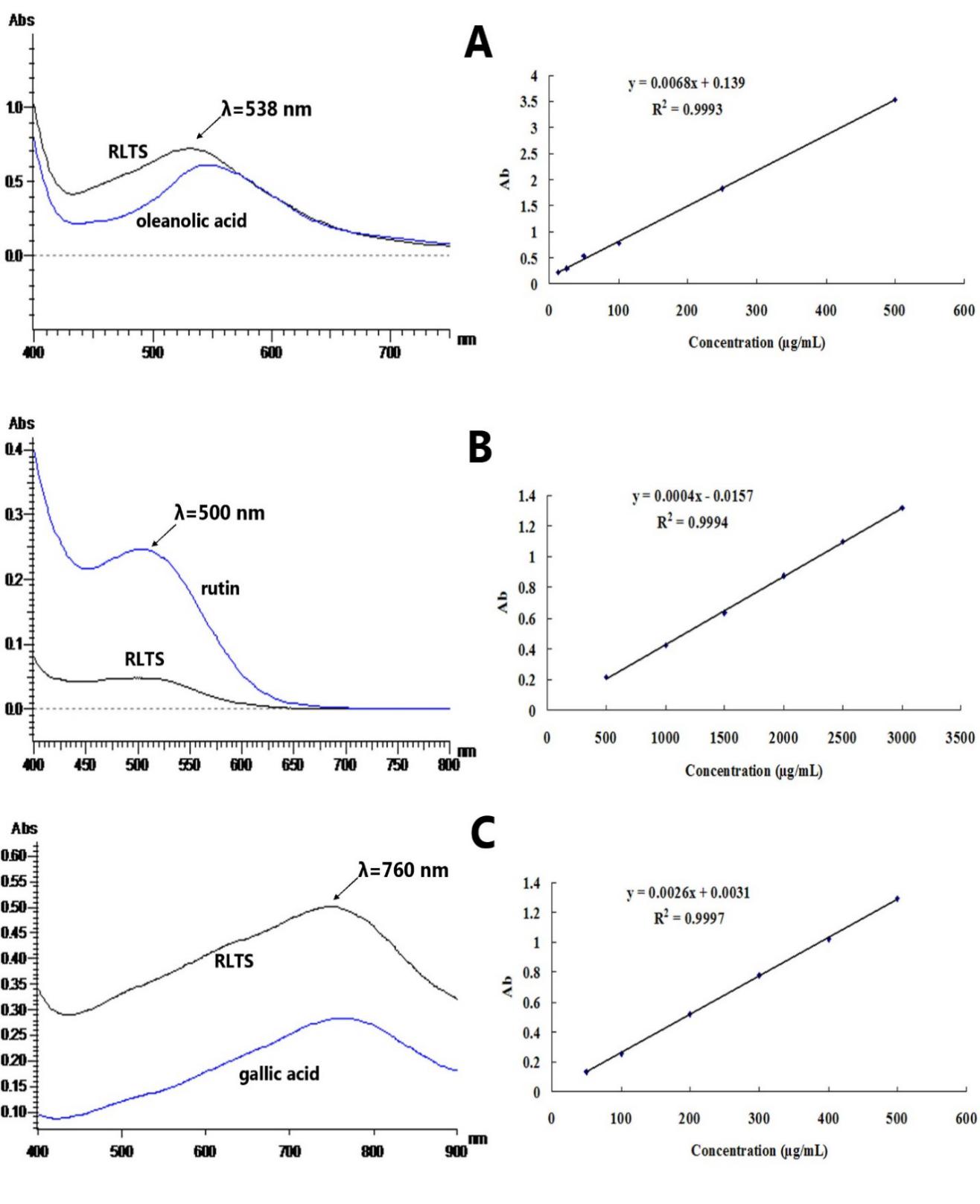


**Figure S1.** Detection wavelengths and calibration curves for the determination of total saponins, total flavonoids and tannin in the extract. Total saponins (**A**); Total flavonoids (**B**); Tannin (**C**).
